# Eradication therapy for *Helicobacter pylori* infection improves nutrition status in Japanese hemodialysis patients: a pilot study

**DOI:** 10.3164/jcbn.18-61

**Published:** 2018-10-02

**Authors:** Hitomi Ichikawa, Mitsushige Sugimoto, Yukitoshi Sakao, Shu Sahara, Naro Ohashi, Koji Sano, Shigeru Tadokoro, Hisanori Azekura, Akira Shimomura, Fuyuki Yamashita, Daiki Sugiyama, Ken Fukuta, Takahisa Furuta, Akihiko Kato, Ken Sugimoto, Hideo Yasuda

**Affiliations:** 1 First Department of Medicine, Hamamatsu University School of Medicine, 1-20-1 Handayama, Higashi-ku, Hamamatsu, Shizuoka 431-3192, Japan; 2 Division of Digestive Endoscopy, Shiga University of Medical Science Hospital, Seta, Tsukinowa-cho, Otsu, Shiga 520-2192, Japan; 3 Hamana Clinic, 235-1 Numa, Hamakita-ku, Hamamatsu, Shizuoka 434-0037, Japan; 4 Sano Clinic, 1818 Tennou-cho, Higashi-ku, Hamamatsu, Shizuoka 435-0052, Japan; 5 Tadokoro Clinic, 1239 Uchino, Hamakita-ku, Hamamatsu, Shizuoka 434-0044, Japan; 6 Sanaru Sun Clinic, 2-14-39 Higashiiba, Naka-ku, Hamamatsu, Shizuoka 432-8036, Japan; 7 Sanarudai Asahi Clinic, 5-20-10 Sanarudai, Naka-ku, Hamamatsu, Shizuoka 432-8021, Japan; 8 Yamashita Clinic, 2-1-5 Nakaizumi, Iwata, Shizuoka 438-0078, Japan; 9 Satsuki no Mori Clinic, 1665-2 Nakase, Hamakita-ku, Hamamatsu, Shizuoka 434-0012, Japan; 10 Hiryu Clinic, 304-9 Akura, Futamata-cho, Tenryu-ku, Hamamatsu, Shizuoka 431-3311, Japan; 11 Center for Clinical Research, Hamamatsu University School of Medicine, 1-20-1 Handayama, Higashi-ku, Hamamatsu, Shizuoka 431-3192, Japan; 12 Blood Purification Unit, Hamamatsu University School of Medicine, 1-20-1 Handayama, Higashi-ku, Hamamatsu, Shizuoka 431-3192, Japan

**Keywords:** ghrelin, nutritional marker, eradication, *Helicobacter pylori*, hemodialysis

## Abstract

Plasma ghrelin level is influenced by *Helicobacter pylori* (*H. pylori*) status and the severity of gastric mucosal atrophy, and the ghrelin level is associated with nutrition status in hemodialysis patients. Here, we investigated the efficacy of *H. pylori* eradication therapy in improving nutrition status in relation to the ghrelin level in *H. pylori*-positive hemodialysis patients. Of *H. pylori*-positive patients receiving hemodialysis at 8 dialysis center, 21 patients underwent gastroduodenoscopy for evaluation of the severity of gastric atrophy, and nutrition markers and plasma ghrelin levels before and 1 year after *H. pylori* eradication therapy were evaluated. Serum cholinesterase level was significantly increased after *H. pylori* eradication compared with the level before eradication (303.2 ± 76.0 vs 287.3 ± 68.1 IU/L, *p* = 0.029). In particular, cholesterol (before, 196.6 ± 23.2 mg/dl; after, 206.1 ± 25.9 mg/dl, *p* = 0.042) and cholinesterase levels (before, 296.9 ± 70.8 IU/L; after, 316.4 ± 73.8 IU/L, *p* = 0.049) increased more strongly in patients with mild–moderate atrophy than those with severe atrophy, irrespective of improvement of plasma acyl-ghrelin and desacyl-ghrelin levels after eradication therapy. In conclusion, *H. pylori* eradication may improve nutrition status by increasing serum cholinesterase and cholesterol levels in hemodialysis patients, especially those with mild and moderate gastric mucosal atrophy.

## Introduction

The state of metabolic and nutritional derangement, called protein-energy wasting (PEW), has a major impact on mortality in hemodialysis patients.^(1,2)^ In general, PEW causes severe and complex processes of muscle loss, poor food intake, inflammation, and cardiovascular disease pathways.^(3)^ Improving the prognosis of hemodialysis patients with PEW therefore requires clarification of the biological mechanism of PEW development and the establishment of countermeasures against the development of PEW.

Ghrelin, an orexigenic peptide produced mainly by X/A-like cells in the stomach, is a major factor associated with malnutrition in general patients and with the pathogenesis of PEW in hemodialysis patients.^(3–5)^ Ghrelin has multiple favorable functions, including enhancement of orexigenic effect, protein anabolism, anti-inflammatory actions, a sensor of systemic oxidative stress, increase of preprandial acid secretion and cardiovascular protection.^(5–9)^ Recently, in addition, ghrelin is shown to stimulate food intake and to play gastric motility via expression of gastric ghrelin-induced c-kit protein in patients with functional dyspepsia and animal model.^(10–13)^ Hemodialysis patients with a low ghrelin level are at increased risk of cardiovascular mortality,^(14)^ and recent clinical trials show that administration of ghrelin improves malnutrition by increasing appetite and food intake.^(15,16)^ Therefore, both nutrition status and prognosis in hemodialysis patients are correlated with ghrelin levels.

*Helicobacter pylori* (*H. pylori*) infection affects the incident rate of gastroduodenal disease and nutritional status.^(17,18)^ *H. pylori* eradication therapy often causes individuals with nomal renal function to develop hyperlipidemia and hyperproteinaemia, along with an increase of body weight and body mass index (BMI).^(19)^ This phenomenon is considered to be due to increases in plasma ghrelin level followed by increased appetite and food intake after *H. pylori* eradication therapy.^(20,21)^ In general, ghrelin is down-regulated by *H. pylori* infection and up-regulated by *H. pylori* eradication therapy. Biological roles of gastric and plasma ghrelin are altered in response to *H. pylori* infection and according to time-course expression levels of ghrelin are decreasing in relation with severity of gastric mucosal atrophy.^(22)^ Previously, we reported that plasma ghrelin level was significantly lower in hemodialysis patients with *H. pylori* infection than in those without this condition, and was inversely correlated with the severity of gastric mucosal atrophy in *H. pylori*-positive patients.^(23)^ We hypothesized that ghrelin level increases after eradication therapy and that nutritional status and prognosis are improved in hemodialysis patients after *H. pylori* eradication.

In this study, we clarified infection rates of *H. pylori* in Shizuoka, Japan, and investigated plasma ghrelin levels and nutritional markers before and after *H. pylori* eradication therapy in *H. pylori*-positive Japanese hemodialysis patients.

## Materials and Methods

Approval for the study protocol was provided by the Institutional Review Board of Hamamatsu University School of Medicine (Number: 23-185) and all patients provided written informed consent before enrollment. The registration number of this clinical research was University hospital Medical Information Network (UMIN) 000023336.

### Subjects

The study enrolled patients receiving maintenance hemodialysis at 8 dialysis centers in Shizuoka, Japan (Sano Clinic, Tadokoro Clinic, Sanaru Sun Clinic, Sanarudai Asahi Clinic, Yamashita Clinic, Satsuki no Mori Clinic, Hiryu Clinic and Hamana Clinic). All patients had received regular hemodialysis for 4 h three times per week for at least 1 month. Exclusion criteria were a history of *H. pylori* eradication, history of gastrectomy, significant clinical illness (e.g., cancer), lack of informed consent and non-dialysis.

Hemodialysis patients who were serologically positive for *H. pylori* (anti-*H. pylori* antibody titer of more than 10) and provided informed consent for *H. pylori* eradication therapy underwent gastroduodenoscopy before eradication therapy. Blood samples were taken for measurement of plasma acyl- and desacyl-ghrelin levels, nutrition markers (serum albumin, total cholesterol and cholinesterase) and an inflammation marker [serum C-reactive protein (CRP)].

Eradication of *H. pylori* was performed by triple therapy with esomeprazole 20 mg, amoxicillin 250 mg or 750 mg, and clarithromycin 200 mg twice daily for 1 week. After therapy, success of eradication was confirmed by the ^13^C-urea breath test conducted at least 4 weeks after the end of eradication therapy.

One year after confirmed eradication, patients underwent gastroduodenoscopy and blood samples were taken for measurement of ghrelin, nutrition markers and CRP. As an index of nutritional status, we calculated the geriatric nutritional risk index (GNRI).^(24,25)^ If the dry weight after hemodialysis is higher than the ideal body weight, we calculated GNRI with dry weight/ideal body weight as 1.^(24,25)^

### Assay of plasma ghrelin levels

Blood samples were taken from all fasted patients in the morning. Samples were collected in an aprotinin/EDTA-containing tube and immediately centrifuged at 1,500 × *g* for 15 min at 4°C. The resulting plasma was agitated with the addition of 1 mol/L hydrochloric acid equal to one-tenth of the plasma volume and stored at –80°C until assay. We used a two-site sandwich enzyme-linked immunosorbent assay specific for acyl-ghrelin and desacyl-ghrelin (Active Ghrelin ELISA Kit and Des-acyl Ghrelin ELISA Kit; SCETI, Tokyo, Japan).

### Diagnosis of *H. pylori* infection and assessment of gastric mucosal atrophy

*H. pylori* infection was diagnosed using an anti-*H. pylori* IgG serological test (E plate Eiken *H. pylori* antibody; Eiken Chemical Co. Ltd., Tochigi, Japan). Patients were considered positive when the anti-*H. pylori* antibody titer was more than 10. The endoscopic gastric mucosal atrophic pattern was evaluated according to the Kimura–Takemoto classification.^(26)^ Severity of atrophy was categorized into the four groups of non-atrophic; mild, Close (C)-I and C-II; moderate, C-III and Open (O)-I; and severe, O-II and O-III.

### Assay of serum pepsinogen levels

Serum levels of pepsinogen (PG) I and PG II were measured using a commercially available kit (Pepsinogen CLEIA; Fuji Rebio Ltd., Tokyo, Japan) by chemiluminescence enzyme immunoassay (EIA), and PG I/II ratio was calculated as a serological marker of gastric mucosal atrophy.^(27)^

### Statistical analyses

Normally numerical values were expressed as mean ± SD. The comparison of clinical parameters before and after *H. pylori* eradication therapy was assessed using paired *t* test or one-way ANOVA. Categorical variables were compared using the Chi-square test. All *p* values were two-sided. *P*<0.05 was considered statistically significant. Calculations were carried out using StatView 5.0 statistical software (SAS Institute, Cary, NC).

## Results

### Patient characteristics

Of Japanese hemodialysis patients in this multicenter study, 14.7% (95% CI: 11.4–18.5%) were serologically positive for *H. pylori*. Eradication rate by triple therapy with esomeprazole 20 mg, amoxicillin 250 mg or 750 mg, and clarithromycin 200 mg twice daily for 1 week was 81.1% (95% CI: 64.8–92.0%). Of the 25 patients who agreed to this follow-up survey, 4 patients dropped out, due to relocation (*n* = 1), loss to follow-up (*n* = 1) or study withdrawal (*n* = 2). Finally, 21 hemodialysis patients were evaluated for change in serological nutrition markers and plasma ghrelin level at 1 year after *H. pylori* eradication therapy. Hemodialysis patients with failured eradication therapy were received the second-line therapy (esomeprazole, amoxicillin, and metronidazole) for 7 days.

The mean age of hemodialysis patients was 64.4 ± 9.5 years, mean body weight (dry weight) was 57.9 ± 14.5 kg, and mean BMI was 22.6 ± 4.6 kg/m^2^ (Table 1). The causes of end-stage renal disease were chronic glomerulonephritis (*n* = 10), diabetic nephropathy (*n* = 5), nephrosclerosis (*n* = 2), IgA nephropathy (*n* = 1) and others (*n* = 3). The mean duration of hemodialysis was 4.7 ± 4.5 years. Endoscopy revealed no peptic ulcer lesions or gastric cancer in any patient. In contrast, all patients had gastric mucosal atrophy with diffuse redness. The severity of gastric mucosal atrophy before eradication therapy was mild (3/21, 14.3%), moderate (5/21, 23.8%) and severe (13/21, 61.9%).

### Influence of *H. pylori* eradication therapy on plasma ghrelin level

Plasma acyl- and desacyl-ghrelin levels before eradication were 11.6 ± 8.6 fmol/ml and 194.9 ± 117.1 fmol/ml (Table 1) versus 9.7 ± 5.1 fmol/ml and 223.3 ± 140.8 fmol/ml after eradication, respectively. No significant differences between plasma acyl- and desacyl-ghrelin levels before and after eradication were observed (*p* = 0.274 and *p* = 0.455, respectively) (Table 1).

### Influence of *H. pylori* eradication therapy on nutrition markers

Although there were no significant changes in dry weight or BMI before and after eradication, serum cholinesterase level after eradication was significantly higher than that before eradication (303.2 ± 76.0 IU/L vs 287.3 ± 68.1 IU/L, *p* = 0.029) (Table 1).

Because plasma ghrelin level is associated with the severity of gastric mucosal atrophy,^(23)^ we evaluated the relationship between nutrition markers and severity of gastric mucosal atrophy to clarify the characteristics of patients whose nutrition markers improved. While nutrition markers (serum total cholesterol, cholinesterase, albumin and GNRI) in patients with severe atrophy did not improve, serum cholesterol levels (before, 196.6 ± 23.2 mg/dl; after, 206.1 ± 25.9 mg/dl, *p* = 0.042) and cholinesterase levels (before, 296.9 ± 70.8 IU/L; after, 316.4 ± 73.8 IU/L, *p* = 0.049) were significantly increased after eradication in patients who had mild to moderate atrophy (Table 2).

### Serum pepsinogen levels in hemodialysis patients after eradication therapy

**Fig. 1:**
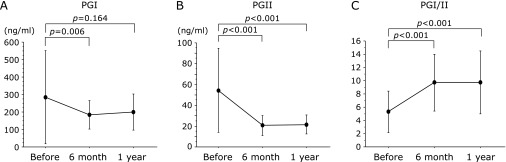
Serum pepsinogen (PG) I level (A), serum PG II level (B) and PG I/II ratio (C) before and after eradication. Serum PG II levels were significantly decreased and PG I/II ratio was significantly increased after eradication.

Serum PG I and PG II levels and PG I/PG II ratio were 199.5 ± 102.3 ng/ml, 21.5 ± 9.2 ng/ml and 9.8 ± 4.8, respectively. The serum PG II level was significantly decreased (before, 54.4 ± 40.3 ng/ml; after, 21.5 ± 9.2 ng/ml, *p*<0.001) and PG I/II ratio was significantly increased after eradication (before 5.3 ± 3.1, after 9.8 ± 4.8, *p*<0.001) (Table 1 and Fig. 1).

## Discussion

We demonstrated that the serological markers, cholinesterase and cholesterol, increased after *H. pylori* eradication therapy in *H. pylori*-positive hemodialysis patients, especially in those with gastric mild–moderate atrophy who has ability for recovery of gastric mucosal atrophy after eradication therapy.^(28)^ Therefore, eradication therapy might improve nutritional status in *H. pylori*-postive hemodialysis patients. Nutritional status is expected to have a markedly greater effect on prognosis in hemodialysis patients than in individuals with nomal renal function. We therefore recommend that hemodialysis patients**be checked for *H. pylori* infection and that eradication therapy should be initiated before the progression of gastric atrophy.

### Association between *H. pylori* eradication and ghrelin levels in hemodialysis patients

*H. pylori* infection down-regulated plasma and gastric mucosal ghrelin levels, and gastric ghrelin mRNA level and number of ghrelin-positive cells significantly increased after eradication.^(22,23,29,30)^ Although it has remained unclear whether ghrelin levels significantly increased after eradication therapy in hemodilysis patients, increasing appetite and food intake was observed in hemodialysis patients with successful eradication. However, we failed to show that plasma ghrelin levels and acyl-ghrelin/total ghrelin ratio after eradication therapy increased compared with before therapy.^(31)^ Because plasma ghrelin levels were reported to have no correlations with expression of gastric ghrelin mRNA or the number of ghrelin-positive cells after eradication therapy,^(29)^ improvement of nutrition markers may cause by increase of gastric ghrelin mRNA and the number of ghrelin-positive cells might increase after eradication therapy. However, we have no sample to measure gastric ghrelin levels and this is limitation of this study. Therefore, we should seek to clarify whether the gastric ghrelin level changes after *H. pylori* eradication therapy in hemodialysis patients.

### Association between *H. pylori* eradication and nutrition markers

Nutrition status is regulated by hormonal systems such as ghrelin and leptin.^(32)^ Chronic gastritis affects a variety of endocrine functions of the stomach including the production of the gastrointestinal hormones and neurotransmitters somatostatin, gastrin and ghrelin.^(10–12)^ Patients often develop hyperlipidemia after eradication therapy and their body weight and BMI increase,^(19,33–35)^ suggesting that *H. pylori* is involved in the physiologic regulation of ghrelin and leptin.^(36)^ PEW is defined as a state of decreased body stores of protein and energy fuels (body protein and fat mass) and is diagnosed if the following three features are present: (1) abnormal nutrition markers (i.e., low serum levels of albumin or cholesterol); (2) reduced body mass (i.e., low or reduced body or fat mass or weight loss with reduced intake of energy); and (3) reduced muscle mass (i.e., muscle wasting or sarcopenia, and reduced mid-arm muscle circumference).^(37)^ In this study, we observed an increase of cholinesterase and cholesterol levels after eradication. Because serum cholesterol level is included in the diagnostic criteria for PEW as a nutrition marker, the increase in cholesterol level after eradication might contribute to improved prognosis in hemodialysis patients. It is possible that the improvement in nutrition status and increase in BMI after eradication therapy might depend not only on an increase of ghrelin but also on another biological mechanisms. Such mechanisms include an increase of gastrointestinal motility,^(10,11,38)^ change in gut microbiome profile,^(39)^ and increase in absorption ability after eradication.^(40)^ Zhang *et al.*^(38)^ showed that in patients with functional dyspepsia, gastric half-emptying times in *H. pylori*-negative patients and eradicated patients were significantly shorter than those in patients who underwent conventional treatment. Betrapally *et al.*^(39)^ reported that alterations to the intestinal microbiota affect the development of nonalcoholic steatohepatitis by influencing digestion, development of obesity, the immune response, and production of gut hormones. *H. pylori* eradication therapy changed the gastrointestinal microbiota.^(41)^ Therefore, eradication therapy might have improved nutrition status. A study to examine whether the long-term prognosis of hemodialysis patients is improved by eradication therapy will be required to investigate this hypothesis further.

## Conclusion

In this small preliminary study, *H. pylori* eradication did increase levels of nutritional markers in patients with mild and moderate gastric atrophy. *H. pylori* eradication therapy might therefore contribute not only to the prevention of gastrointestinal disease, but also to improvements in the prognosis of hemodialysis patients. We recommend that the *H. pylori *status of hemodialysis patients be checked. We plan to conduct a long-term follow-up survey to investigate whether the prognosis of *H. pylori*-positive hemodialysis patients improves after eradication; and if so, whether this improvement is related to plasma ghrelin level and nutritional markers.

## Author Contributions

Ichikawa H, Sugimoto M, Sakao Y, Sahara S, Ohashi N and Yasuda H contributed to study conception and design; Ichikawa H, Sakao Y, Sahara S, Ohashi N, Sano K, Tadokoro S, Azekura H, Shimomura A, Yamashita F, Sugiyama D and Fukuta K contributed to data acquisition; Ichikawa H, Sugimoto M, Sakao Y and Ohashi N contributed to data analysis and interpretation; Ichikawa H and Sugimoto M wrote the paper; Kato A, Sugimoto K and Furuta T edited the paper.

## Figures and Tables

**Table 1:** Characteristics and nutrition markers of hemodialysis patients before and after eradication therapy

	Before eradication	After eradication	*p* value
Body weight (kg)	57.9 ± 14.5	57.8 ± 15.2	0.715
Body mass index (kg/m^2^)	22.6 ± 4.6	22.5 ± 4.8	0.566
Albumin (g/dl)	4.0 ± 0.3	4.1 ± 0.3	0.063
Total cholesterol (mg/dl)	187.3 ± 37.2	193.8 ± 38.5	0.118
Cholinesterase (IU/L)	287.3 ± 68.1	303.2 ± 76.0	0.029
GNRI	90.6 ± 7.7	92.1 ± 7.6	0.055
CRP (mg/dl)	0.4 ± 0.8	0.2 ± 0.2	0.469
Acyl-ghrelin (fmol/ml)	11.6 ± 8.6	9.7 ± 5.1	0.274
Desacyl-ghrelin (fmol/ml)	194.9 ± 117.1	223.3 ± 140.8	0.455
Acyl-ghrelin/total ghrelin	5.5 ± 2.5	4.7 ± 1.8	0.244
Pepsinogen I (ng/ml)	285.8 ± 266.0	199.5 ± 102.3	0.164
Pepsinogen II (ng/ml)	54.4 ± 40.3	21.5 ± 9.2	<0.001
Pepsinogen I/II ratio	5.3 ± 3.1	9.8 ± 4.8	<0.001

Values are presented as mean ± SD. *P* value: before vs 1 year after eradication. GNRI, geriatric nutritional risk index; CRP, C-reactive protein.

**Table 2:** Change in nutrition parameters after *H. pylori* eradication according to severity of gastric mucosal atrophy

Severity of gastric mucosal atrophy	Mild/Moderate	Severe
Age (years)	62.9 ± 8.8	65.3 ± 10.1
Male/Female (*n*/*n*)	5/3	6/7
Duration of hemodialysis (years)	4.6 ± 5.0	4.7 ± 4.4

	Before	After	*p* value	Before	After	*p* value
Body weight (kg)	61.7 ± 15.2	61.2 ± 15.7	0.575	55.6 ± 14.2	55.7 ± 15.2	0.944
Body mass index (kg/m^2^)	23.3 ± 4.3	23.1 ± 4.6	0.575	22.2 ± 4.9	22.2 ± 5.1	0.753
Albumin (g/dl)	4.0 ± 0.3	4.1 ± 0.4	0.363	4.0 ± 0.3	4.1 ± 0.3	0.093
Total cholesterol (mg/dl)	196.6 ± 23.2	206.1 ± 25.9	0.042	181.6 ± 43.5	186.2 ± 43.8	0.6
Cholinesterase (IU/L)	296.9 ± 70.8	316.4 ± 73.8	0.049	281.4 ± 68.6	295.2 ± 79.1	0.182
GNRI	91.4 ± 7.9	92.5 ± 6.5	0.363	90.0 ± 7.8	91.9 ± 8.4	0.093
CRP (mg/dl)	0.2 ± 0.3	0.2 ± 0.1	0.866	0.5 ± 1.0	0.2 ± 0.3	0.272
Acyl-ghrelin (fmol/ml)	9.8 ± 8.5	9.1 ± 4.7	0.889	12.7 ± 8.8	10.1 ± 5.5	0.221
Desacyl-ghrelin (fmol/ml)	184.0 ± 131.5	252.9 ± 201.2	0.575	201.6 ± 112.4	205.1 ± 92.0	0.65
Acyl-ghrelin/total ghrelin	5.0 ± 2.2	4.4 ± 1.8	0.263	5.8 ± 2.6	4.9 ± 1.7	0.463

GNRI, geriatric nutritional risk index; CRP, C-reactive protein.

## Abbreviations

BMI: body mass index
*H. pylori*: *Helicobacter pylori*
PEW: protein-energy wasting
PG: pepsinogen
